# A comprehensive evaluation of drought resistance in *Hemerocallis fulva* L. using membership function and principal component analysis

**DOI:** 10.1038/s41598-025-18700-9

**Published:** 2025-10-06

**Authors:** Zheng Liang, Ke Pei, Hongliang Zhang, Xiaoyi Lai, Yiran Meng, Minlong Jia, Dongmei Cao, Chao Zhang, Zhuoqin Song, Jiuju Duan

**Affiliations:** 1 College of Horticulture, Shanxi Agricultural University, Taiyuan, 030031 China; 2 College of Architecture and Design, Shanxi Vocational University of Engineering Science and Technology, Jinzhong, 030619 China

**Keywords:** Drought resistance, *Hemerocallis fulva*, Principal component analysis, Membership function analysis, Cluster analysis, Plant physiology, Drought

## Abstract

Drought is a significant environmental stressor affecting a broad range of ornamental plants. Selecting and developing drought-resistant ornamental plants is a key strategy to solve this problem. This study comprehensively evaluated the drought resistance of 25 *Hemerocallis fulva* genotypes, which were collected from natural populations along the Taihang Mountain range in Shanxi Province, China. Five growth indices and eight physiological parameters were measured both prior to and following drought stress exposure in plants. These parameters included total chlorophyll (Chl) content, Chl *a* and Chl *b* contents, the Chl *a/b* ratio, carotenoid content, leaf relative water content (RWC), electrolyte leakage, and malondialdehyde (MDA) content. The results indicated that drought stress led to a reduction in plant height, canopy width, as well as leaf length, width, and area. Interestingly, under drought conditions, the contents of total Chl, Chl *a*, Chl *b*, and carotenoids, along with the Chl *a/b* ratio, generally increased, though the extent of these changes varied across different genotypes. On the other hand, the drought treatment resulted in elevated electrolyte leakage and MDA content, while RWC showed a marked decrease. To better understand the underlying physiological responses, Principal Component Analysis (PCA) was applied to condense the thirteen individual growth and physiological parameters into three principal components: (1) a plant growth index, (2) a photosynthetic pigments index, and (3) a comprehensive water status and cell membrane damage index. Based on the comprehensive evaluation scores, cluster analysis, and heatmap analysis, the 25 *Hemerocallis fulva* wild genotypes were categorized into three groups: high drought-resistance, moderate drought-resistance and low drought-resistance. Genotypes Taiyuan No.1, Taiyuan No.2, Qinshui No.1, Taigu No.1, Jiaocheng No.1 and Datong No.1 were classified as exhibiting high drought resistance. No significant correlation was observed between drought resistance and either chromosome ploidy or the number of flower petals in wild *Hemerocallis fulva*. Drought resistance showed a positive correlation with the Chl *a/b* ratio and a negative correlation with leaf electrolyte leakage. This study not only provides valuable genetic resources for landscape architecture in arid regions. Moreover, it establishes a comprehensive and reliable methodology for evaluating drought resistance across different *Hemerocallis fulva* genotypes.

## Introduction

Drought, recognized as one of the most critical climatic events, significantly impacts ecosystems, productive activities, and human livelihoods^1,2^. It is also the most extensive and devastating disaster in terms of agricultural losses^3^. Over the past few decades, this situation has become increasingly severe, threatening global food security. In 2020, global drylands accounted for approximately 40.6% of the Earth’s terrestrial surface area (excluding Antarctica)^4^. As the global climate continues to warm, it is anticipated that more regions will experience exacerbated drought conditions in the future^5,6^.

Ornamental plants are widely popular for their significant aesthetic appeal and their role in improving the environment. Integrating ornamental plants into urban green spaces is widely acknowledged as an effective approach to mitigating urbanization challenges, including pollution and the heat island effect. However, their cultivation traditionally requires substantial water resources for irrigation^7^. Given that approximately 25% of urban water resources are allocated to watering landscapes and gardens, water scarcity may present challenges in sustaining green spaces^8^. Although specialized agronomic measures can be employed during periods of drought, the selection and cultivation of drought-resistant plant varieties undoubtedly provide more sustainable ecological and economic advantages^9^. To adapt to dry conditions, drought- resistant plants exhibit a suite of adaptive mechanisms to alleviate stress, which include the active accumulation of compatible solutes, osmotic adjustment, augmentation of root-to-shoot ratios, growth retardation, and modifications in leaf anatomical structures^10–12^. It is essential to understand the mechanisms underlying plant responses to water deficiency and to develop drought-resistant varieties.

*Hemerocallis fulva* is highly valued for its culinary, medicinal, and aesthetic properties^13–15^. As one of the important plant species in urban landscaping, *Hemerocallis fulva* have been extensively utilized due to its diverse flower colors, shapes, and climate adaptability^16–18^. According to the American *Hemerocallis* Association, over 100,000 varieties of this plant have been developed^9^. The drought-resistant characteristics of *Hemerocallis fulva* make it an effective means of conserving both manpower and water resources. Consequently, selecting varieties with high drought resistance of *Hemerocallis fulva* has been one of the important factors to consider in the allocation of plants for urban landscaping.

The drought resistant characteristics of plants is a complex biological process, influenced by factors such as genotype, the intensity and duration of stress, and developmental stage. The plant’s response to drought stress involves interactions among various physiological and biochemical parameters. Given the diversity of drought resistance parameters, a comprehensive analysis of multiple factors is necessary for accurately evaluating the drought tolerance capabilities of various cultivars. Principal Component Analysis (PCA), a factor analysis method, transforms multiple variables into a smaller set of uncorrelated comprehensive indicators^19,20^. The membership function method employs the weighted average method (D-value) to address the limitations of individual indicators^21^. PCA and membership function method can be extensively utilized to evaluate ornamental plant drought resistance in *Paeonia lactiflora*^21^, *Iris germanica*^22^ and red fescue cultivars^23^.

In this study, we determined the variations in 13 morphological and physiological traits across 25 wild genotypes of *Hemerocallis fulva* from the Taihang mountain areas in China under drought conditions. We applied PCA, membership function analysis, and clustering analysis to comprehensively evaluate and classify their drought tolerance, with the aim of identifying genotypes with superior drought adaptation mechanisms. The drought-resistant genotypes identified in this study may serve as valuable genetic resources for the incorporation of drought-resistant traits into new cultivars, enhancing the resilience of *Hemerocallis fulva* to water scarcity. They can also be preferentially applied to arid regions for landscaping applications. Additionally, the method employed for evaluating drought resistance in *Hemerocallis fulva* can serve as a reference for assessing drought tolerance in other plant species.

## Materials and methods

### Collection of germplasm

**Fig. 1:**
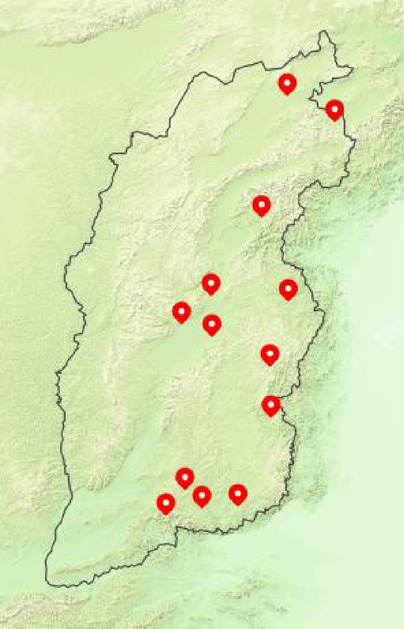
Material collection counties along the Taihang Mountains. The map is sourced from National Platform for Common GeoSpatial Information Services (www.tianditu.gov.cn). Edit with Di Map Editor v2.0.2-alpha.27 (www.ditushu.com).

The germplasm of 25 *Hemerocallis fulva* genotypes (Table 1) were collected from multiple natural habitats occurring in different localities in 13 counties along the Taihang Mountain range in Shanxi Province, China (Fig. 1). They were identified by *Hemerocallis* research team of Shanxi Agricultural University^24^, and preserved in Shanxi Herbaceous Ornamental Plant Germplasm Resource Nursery (Shanxi, China) with the corresponding accession numbers provided in Table 1. The genotypes utilized in this study were acquired in compliance with applicable guidelines, and no specific permissions were required.

**Table 1:** List of 25 *Hemerocallis fulva* genotypes used in the present study.

Material No	Material name	Chromosom number and ploidy	Sources of genotypes	Petal type	Accession No
S1	Taiyuan No.1	2n = 3x = 33	Taiyuan City	Double	SXHH0000024
S2	Wutai No.2	2n = 3x = 33	Wutai County	Double	SXHH0000021
S3	Pingding No.1	2n = 2x = 22	Pingding County	Single	SXHH0000008
S4	Taiyuan No.2	2n = 2x = 22	Taiyuan City	Single	SXHH0000010
S5	Zuoquan No.1	2n = 2x = 22	Zuoquan County	Single	SXHH0000002
S6	Lishan No.1	2n = 3x = 33	Lishan Nature Reserve	Single	SXHH0000025
S7	Guangling No.2	2n = 2x = 22	Guangling County	Single	SXHH0000013
S8	Qinshui No.1	2n = 2x = 22	Qinshui County	Single	SXHH0000001
S9	Zezhou No.2	2n = 2x = 22	Zezhou County	Single	SXHH0000012
S10	Taigu No.1	2n = 3x = 33	Taigu County	Single	SXHH0000023
S11	Jiaocheng No.4	2n = 3x = 33	Jiaocheng County	Single	SXHH0000027
S12	Licheng No.3	2n = 3x = 33	Licheng County	Double	SXHH0000017
S13	Jiaocheng No.3	2n = 2x = 22	Jiaocheng County	Single	SXHH0000011
S14	Zezhou No.1	2n = 3x = 33	Zezhou County	Double	SXHH0000022
S15	Licheng No.2	2n = 2x = 22	Licheng County	Single	SXHH0000007
S16	Licheng No.6	2n = 2x = 22	Licheng County	Single	SXHH0000004
S17	Yangcheng No.1	2n = 3x = 33	Yangcheng County	Double	SXHH0000016
S18	Jiaocheng No.2	2n = 2x = 22	Jiaocheng County	Single	SXHH0000009
S19	Jiaocheng No.1	2n = 3x = 33	Jiaocheng County	Single	SXHH0000026
S20	Datong No.1	2n = 3x = 33	Yunzhou District of Datong City	Single	SXHH0000353
S21	Jiaocheng No.5	2n = 3x = 33	Jiaocheng County	Double	SXHH0000028
S22	Wutai No.1	2n = 2x = 22	Wutai Mountain Scenic Area	Single	SXHH0000014
S23	Licheng No.1	2n = 3x = 33	Licheng County	Double	SXHH0000020
S24	Licheng No.4	2n = 3x = 33	Licheng County	Single	SXHH0000018
S25	Licheng No.5	2n = 2x = 22	Licheng County	Single	SXHH0000003

### Conditions of cultivation

This research was conducted at the College of Horticulture, Shanxi Agricultural University, Shanxi, China. Three-year-old seedlings were transplanted to plastic pots (20 × 30 cm) filled with garden soil. Each pot contained 3 plants. All plants were grown in the greenhouse with temperatures ranging from 23 to 27 °C, humidity between 30 and 50%, and a light intensity of 25,000 Lux. Two months later, healthy and vigorous plants of equal size were selected as materials for the experiment.

### Drought treatments

The experimental design employed a randomized block design with two treatments: full irrigation for the control group (Control) and deficit irrigation for the drought treatment group (Treatment). The control group was irrigated normally to maintain the soil water content at 75–80% of the maximum water holding capacity (MWHC). The drought treatment groups were subjected to natural drought with no watering. After 28 days of drought treatment, the soil water content decreased to 25–30% of MWHC, which was in the range of severe stress. Each treatment involved ten plants of each genotype, and was repeated three times. The center leaves of plants were collected after 0, 7, 14, 21 and 28 days of drought stress as test materials. Samples from three independent plants were used as individual replicates for testing chlorophyll content, leaf relative water (RWC) content, and malondialdehyde (MDA) content. For each sample, three technical replicates were required.

### Measurement of electrolyte leakage (EL)

Electrolyte leakage from leaf tissues was measured using the electrical conductivity method^25^.

### Measurement of malondialdehyde content

Malondialdehyde (MDA) content was measured using the thiobarbituric acid (TBA) reaction as described by Weng^26^. Approximately 0.15 g of fresh leaves were homogenized in 3 mL of 5% trichloroacetic acid. The homogenate was centrifuged at 10,000 g for 15 min to pellet the debris. 5 mL of 0.5% TBA was added to the supernatant, and the mixture was incubated in a boiling bath at 100 °C for 10 min, followed by rapid cooling on ice. The mixture was then centrifuged at 10,000 g for 5 min. The supernatant was collected, and the absorbance was measured spectrophotometrically at 600 nm, 532 nm and 450 nm. The MDA content of the leaf tissue was calculated using the following equation: MDA content (µmol/g FW) = [6.452 × (A532–A600) − 0.559 × A450] × (V/W) × 1000, where V represents the volume of the supernatant and W represents the weight of the fresh leaf tissue.

### Measurement of chlorophyll and carotenoid content

Fresh leaves (0.2 g) were immersed in 10 ml of a mixture of acetone: ethanol: water = 4.5: 4.5: 1 (v/v/v). After incubation at room temperature for 48 h in the dark, the resulting extract was used to measure chlorophyll quantification. Absorbances at 663 nm (for chlorophyll *a*, Chl* a*), 645 nm (for chlorophyll *b*, Chl* b*) and 440 nm (for carotenoid, Car) were determined using a spectrophotometer. The concentrations of chlorophyll *a* (Chl *a*), chlorophyll *b* (Chl *b*) and carotenoid (Car) were calculated using Arnon’s formula: C = (*A* × *V* × 1000) / (*E* × *l*)^27^. In this formula, C represents the chlorophyll or carotenoid content in g/L, A is the absorbance value of the sample, V is the volume of the sample in mL, E is the molar absorption coefficient of the respective pigment (in L/(mol cm)), and *l* is the optical path length in cm.

### Leaf relative water content assay

Leaf relative water content (RWC) was determined following the method described by Patane et al.^28^ Fresh leaves were cut and weighed to obtain their fresh weight (W_f_). The leaves were then submerged in distilled water for 24 h, after which they were weighed again to obtain the saturated weight (W_t_). Subsequently, the leaves were oven-dried at 70 °C for 3 days until reached a constant weight, and were reweighed to obtain the dry weight (W_d_); The RWC was calculated using the following formula: RWC (%) = [(W_f_ − W_d_)/(W_t_ − W_d_)] × 100%.

### Determination of growth indices

The heights and canopy widths for all plants were measured at each sampling time-point. The length, width and area of the second fully expanded leaf on a plant were measured using a scanner (EPSON EXPRESSION 1680) and image analysis software (WinRHIZO, Regent Instruments, Canada).

### Statistical analysis

All experiments were performed in triplicate. All data were statistically analyzed by using a statistical package SPSS Version 22.0 for Windows. A T-test (two-tailed) was used to determine the significant differences (*P* < 0.05) between drought-treated and control samples. Data from three biological replicates were analyzed, and presented as the mean ± standard deviation (SD) at the 0.05 level of significance.

The fuzzy mathematical membership function formula was utilized to transform the original test data. For the index positively correlated with drought resistance, membership function values were calculated using the formula U(X) = (X − X_min_)/(X_max_ − X_min_). Conversely, for the index negatively correlated with drought resistance, the values were determined by U(X) = 1 − (X − X_min_)/(X_max_ − X_min_), where X represents the index value of *Hemerocallis fulva* genotypes, X_min_ indicates the minimum value, and X_max_ indicates the maximum value of the index. Subsequently, the software SPSS 22.0 was employed to PCA, Pearson correlation analysis, and Cluster analysis using the furthest neighbor method.

Ultimately, eigenvalue proportion of each principal component relative to the total eigenvalue of the extracted components was applied as weights to calculate the comprehensive evaluation value, termed the composite score (F value), which assesses drought resistance in *Hemerocallis fulva* genotypes exposed to drought stress^29^. The F values of different *Hemerocallis fulva* genotypes were computed using the formula F = (λ_1_ × F_1_ + λ_2_ × F_2_ + λ_3_ × F_3_ + … + λ_m_ × F_m_)/(λ_1_ + λ_2_ + λ_3_ + … + λ_m_), where λ_i_ (for i = 1,…,m) denotes the eigenvalues of extracted principal components, and F_1_, F_2_,…, F_m_ are calculated as F_j_ = a_1j_ZX_1_ + a_2j_ZX_2_ … + a_pj_ZX_p_ (for j = 1,…,m), with a_ij_ representing the eigenvector elements and of ZX_1_ , ZX_2_ ,…, ZX_p_ representing the standardized original variable values.

Based on the membership function value and the weight of each index, the drought resistant comprehensive evaluation value (D value) was calculated. This value reflects the drought resistance ability, with higher values indicating stronger drought resistance^29,30^. The formula for calculating D value is as follows:

$${\text{D}} = \sum\limits_{{{\text{j}} = 1}}^{{\text{m}}} {\left( {{\text{R}}({\text{X}}_{{{\text{ij}}}} )*{\text{W}}_{{\text{j}}} } \right)}$$

In the formula, W_j_ was the index weight.

## Results and discussion

### Changes in growth traits in response to drought stress

Drought resistance is a complex quantitative trait affected by both genetic and environmental factors^31^. Plants show various morphological and physio-biochemical changes in response to drought stress, which vary among growth stages, species, and cultivars^32,33^. Before drought injury or death, the variation of morphological and physiological indices can be used to predict the impact of drought stress on plants^34^. In the present experiment, 25 wild genotypes of *Hemerocallis fulva* collected along the Taihang Mountain range in Shanxi Province, China were used as experimental materials. The phenotypic observations of drought stress treatments showed that the growth of 25 *Hemerocallis fulva* genotypes was inhibited to different degrees compared with the control group (Table 2). Visible symptoms of *Hemerocallis fulva* subjected to drought stress include leaf wilting, chlorosis, and a reduction in leaf area. The severity of these symptoms, however, varied among genotypes. Drought-sensitive genotypes exhibited pronounced wilting and leaf curling, while drought-resistant genotypes displayed only mild and delayed symptoms. Initially, leaf curling may serve as an active, water-conserving strategy that reduces radiation load and transpirational water loss under drought conditions. However, with prolonged stress exposure, this response shifts from a protective adaptation to a symptom of cellular injury, becoming a passive consequence of water depletion. In this study, Jiaocheng No. 4(S11), Licheng No. 1(S23), and Yangcheng No. 1(S17) have the largest decrease in plant height, reaching 19.58%, 17.66%, and 17.54% respectively. The largest decreases in canopy width were observed in Jiaocheng No. 1(S19), Licheng No. 4(S24), and Licheng No. 5(S25), with reductions of 17.07%, 16.99%, and 16.15%, respectively. The greatest reductions in leaf length were observed in Jiaocheng No. 4(S11), Jiaocheng No. 3(S13), and Licheng No. 4(S24); while the largest reductions in leaf width were found in Licheng No. 1(S23), Qinshui No. 1(S8), and Taiyuan No. 2(S4).

**Table 2:** Changes of growth indices in 25 *Hemerocallis fulva* genotypes under drought stress.

Genotypes	PH (cm)		CW (cm)		LL (cm)		LW (cm)		LA (cm^2^)	
	Control	Treatment	Control	Treatment	Control	Treatment	Control	Treatment	Control	Treatment
S1	40.4 ± 2.6^a^	40.1 ± 4.1^a^	47.9 ± 3.7^a^	45.2 ± 17.9^a^	43.3 ± 3.8^a^	41.6 ± 2.2^a^	1.9 ± 0.2^a^	1.7 ± 0.1^a^	57.7 ± 5.8^a^	49.6 ± 6.7^a^
S2	36.5 ± 1.2^a^	34.0 ± 1.2^b^	47.1 ± 7.4^a^	42.4 ± 5.5^a^	38.9 ± 8.8^a^	36.2 ± 2.7^a^	1.2 ± 0.2^a^	1.2 ± 0.2^a^	32.5 ± 6.7^a^	29.2 ± 5.6^a^
S3	35.5 ± 2.0^a^	32.9 ± 2.4^a^	45.3 ± 4.7^a^	42.2 ± 5.8^a^	41.7 ± 2.6^a^	41.1 ± 11.2^a^	1.1 ± 0.3^a^	1.0 ± 0.2^a^	33.0 ± 6.1^a^	27.7 ± 1.2^a^
S4	37.4 ± 1.2^a^	36.9 ± 3.8^a^	57.3 ± 6.8^a^	53.0 ± 8.2^a^	42.3 ± 3.3^a^	41.9 ± 12.0^a^	1.7 ± 0.3^a^	1.3 ± 0.3^a^	51.3 ± 11.2^a^	39.1 ± 13.7^a^
S5	41.1 ± 1.6^a^	39.4 ± 3.5^a^	48.3 ± 10.7^a^	43.4 ± 4.6^a^	40.6 ± 3.0^a^	40.0 ± 6.7^a^	1.0 ± 0.1^a^	0.9 ± 0.1^a^	29.1 ± 3.9^a^	26.5 ± 4.6^a^
S6	32.6 ± 3.4^a^	30.4 ± 5.0^a^	50.2 ± 2.2^a^	49.9 ± 5.3^a^	41.3 ± 2.4^a^	41.0 ± 9.3^a^	1.4 ± 0.1^a^	1.4 ± 0.2^a^	41.2 ± 3.1^a^	40.3 ± 14.4^a^
S7	34.6 ± 1.7^a^	31.2 ± 7.8^a^	40.8 ± 3.9^a^	37.3 ± 10.5^a^	39.1 ± 2.7^a^	37.9 ± 8.5^a^	1.1 ± 0.1^a^	0.9 ± 0.2^a^	28.9 ± 5.2^a^	23.0 ± 5.2^a^
S8	40.2 ± 1.4^a^	39.7 ± 5.3^a^	61.9 ± 6.1^a^	58.3 ± 9.8^a^	48.8 ± 11.8^a^	46.0 ± 14.0^a^	1.9 ± 0.1^a^	1.4 ± 0.5^a^	62.7 ± 13.0^a^	48.9 ± 31.1^a^
S9	33.0 ± 1.0^a^	30.1 ± 3.3^a^	55.0 ± 16.3^a^	51.7 ± 16.0^a^	43.3 ± 4.2^a^	42.0 ± 8.0^a^	1.3 ± 0.1^a^	1.1 ± 0.2^a^	39.3 ± 5.5^a^	33.0 ± 7.2^a^
S10	35.0 ± 2.6^a^	33.8 ± 9.8^a^	49.7 ± 5.2^a^	45.1 ± 4.0^a^	38.1 ± 10.4^a^	33.2 ± 2.2^a^	1.6 ± 0.2^a^	1.4 ± 0.1^b^	42.4 ± 13.5^a^	32.6 ± 3.7^a^
S11	25.2 ± 2.5^a^	20.2 ± 3.1^b^	38.5 ± 11.7^a^	35.9 ± 5.1^a^	33.4 ± 2.3^a^	24.1 ± 5.1^b^	1.7 ± 0.1^a^	1.5 ± 0.5^a^	39.5 ± 4.8^a^	26.7 ± 13.1^a^
S12	34.3 ± 2.9^a^	31.4 ± 3.8^a^	47.7 ± 9.7^a^	44.7 ± 8.8^a^	30.4 ± 3.2^a^	28.7 ± 2.3^a^	1.0 ± 0.4^a^	0.9 ± 0.1^a^	22.2 ± 9.8^a^	17.2 ± 2.4^a^
S13	20.4 ± 2.7^a^	19.0 ± 1.2^a^	35.6 ± 10.7^a^	30.7 ± 3.6^a^	28.1 ± 3.2^a^	22.1 ± 3.2^b^	1.4 ± 0.3^a^	1.4 ± 0.1^a^	28.1 ± 8.5^a^	21.6 ± 5.6^a^
S14	26.2 ± 3.7^a^	25.6 ± 6.1^a^	44.9 ± 5.1^a^	37.9 ± 6.6^a^	34.7 ± 8.7^a^	31.3 ± 9.7^a^	1.6 ± 0.2^a^	1.4 ± 0.2^a^	38.2 ± 12.8^a^	31.2 ± 11.9^a^
S15	49.5 ± 2.4^a^	48.6 ± 4.0^a^	45.4 ± 4.3^a^	44.4 ± 3.0^a^	44.7 ± 0.7^a^	43.3 ± 5.3^a^	1.4 ± 0.1^a^	1.3 ± 0.2^a^	44.5 ± 3.8^a^	38.4 ± 10.6^a^
S16	21.5 ± 1.6^a^	20.3 ± 1.8^a^	33.7 ± 3.9^a^	31.7 ± 2.7^a^	29.9 ± 3.1^a^	29.7 ± 1.6^a^	1.2 ± 0.0^a^	1.2 ± 0.2^a^	25.3 ± 2.4^a^	24.6 ± 4.2^a^
S17	25.8 ± 2.1^a^	21.3 ± 2.5^b^	43.7 ± 3.1^a^	43.0 ± 5.0^a^	38.2 ± 1.3^a^	37.8 ± 5.1^a^	1.6 ± 0.1^a^	1.5 ± 0.2^a^	43.5 ± 2.2^a^	39.4 ± 9.3^a^
S18	36.4 ± 2.2^a^	34.5 ± 4.4^a^	44.5 ± 9.8^a^	42.9 ± 2.8^a^	32.4 ± 5.3^a^	31.7 ± 0.9^a^	0.9 ± 0.2^a^	0.7 ± 0.2^a^	21.3 ± 6.8^a^	16.0 ± 3.0^a^
S19	39.0 ± 0.4^a^	38.3 ± 1.8^a^	44.4 ± 8.0^a^	36.8 ± 12.9^a^	43.1 ± 3.8^a^	40.9 ± 13.4^a^	1.8 ± 0.3^a^	1.7 ± 0.5^a^	55.1 ± 8.5^a^	51.8 ± 29.2^a^
S20	57.5 ± 1.5^a^	56.3 ± 2.2^a^	54.7 ± 1.5^a^	52.0 ± 6.6^a^	54.8 ± 5.4^a^	53.6 ± 8.8^a^	1.2 ± 0.3^a^	1.2 ± 0.2^a^	45.8 ± 15.7^a^	43.3 ± 9.4^a^
S21	23.8 ± 1.0^a^	23.3 ± 10.5^a^	42.1 ± 7.0^a^	41.6 ± 13.9^a^	36.9 ± 3.3^a^	32.4 ± 10.9^a^	1.3 ± 0.2^a^	1.0 ± 0.1^b^	33.0 ± 6.7^a^	22.7 ± 10.0^a^
S22	29.4 ± 1.0^a^	29.3 ± 6.4^a^	37.9 ± 2.1^a^	36.8 ± 7.9^a^	34.8 ± 3.0^a^	33.1 ± 11.5^a^	0.9 ± 0.1^a^	0.7 ± 0.1^a^	20.6 ± 2.9^a^	15.8 ± 4.0^a^
S23	23.1 ± 2.0^a^	19.0 ± 3.2^a^	36.8 ± 2.4^a^	32.5 ± 4.0^a^	22.7 ± 1.6^a^	19.6 ± 2.3^a^	0.9 ± 0.2^a^	0.7 ± 0.1^a^	14.7 ± 2.4^a^	9.5 ± 2.3^b^
S24	22.3 ± 1.9^a^	19.3 ± 3.1^a^	38.4 ± 5.9^a^	31.9 ± 5.7^a^	30.6 ± 9.5^a^	25.8 ± 5.2^a^	1.6 ± 0.1^a^	1.5 ± 0.2^a^	35.3 ± 12.5^a^	27.2 ± 9.9^a^
S25	28.1 ± 2.1^a^	27.0 ± 3.2^a^	38.6 ± 6.4^a^	32.3 ± 5.2^a^	34.3 ± 4.0^a^	33.9 ± 4.5^a^	1.6 ± 0.3^a^	1.4 ± 0.4^a^	38.6 ± 10.9^a^	32.4 ± 11.4^a^

The data in the table are the average values of each treatment, the results of physiological indices were expressed as mean ± standard deviation, different letters between control and treatment for each genotype indicate significant differences at *P* < 0.05 levels, through paired sample t-test. PH, plant height; CW, canopy width; LL, leaf length; LW, leaf width; LA, leaf area; the same below.

Leaf area declined under drought stress conditions. The most substantial reduction in leaf area were observed in Licheng No. 1(S23), Jiaocheng No. 4(S11) and Jiaocheng No. 5(S21), with decreases of 35.27%, 32.5%, 31.07%, respectively. Under drought stress conditions, the reduction in leaf area of *Hemerocallis fulva* may helps to achieve stability between the water absorbed by the roots and the water status of various plant parts^35^. This reduction also acts as a drought avoidance strategy by minimizing water loss through transpiration^36^. This was similar to the previous findings, where plant growth and biomass were significantly reduced under drought stress, which may have been due to the reduction in an imbalance in plant water status, and a decrease in the production of photoassimilates^37,38^.

### Changes in physiological parameters in response to drought stress

Environmental fluctuations, particularly abiotic stressors such as drought, extreme temperature variations, and excessive light exposure, significantly perturb chlorophyll metabolism^39^. Although some studies have indicated that drought stress does not affect chlorophyll content, an increase in chlorophyll content has been observed under drought stress in *Bouteloua gracilis*^40^ and *Impatiens walleriana*^41^. An increase in total chlorophyll content was also observed in drought-stressed *Hemerocallis fulva*, though the patterns of change varied across genotypes (Table 3). The genotypes that exhibited the largest increase in total chlorophyll content were Wutai No.1(S22), Qinshui No.1(S8) and Datong No.1(S20), with increases of 45.80%, 40.19% and 39.31% respectively. The increased chlorophyll content could be utilized in the biosynthesis of various chloroplast components, such as osmoprotectants, fatty acids, starch, or other stress-protective compounds^41^.

**Table 3:** Changes of chlorophyll and carotenoid in 25 *Hemerocallis fulva* genotypes under drought stress.

Genotypes	Treatment	Chl (mg g^−1^FW)	Chl *a* (mg g^−1^FW)	Chl *b* (mg g^−1^FW)	Chl *a*/*b*	Car (mg g^−1^FW)
S1	Control	1.224 ± 0.181^a^	0.898 ± 0.139^a^	0.326 ± 0.044^a^	2.749 ± 0.149^a^	0.560 ± 0.046^a^
	Treatment	1.241 ± 0.360^a^	0.926 ± 0.278^a^	0.315 ± 0.084^a^	2.913 ± 0.172^a^	0.593 ± 0.119^a^
S2	Control	1.218 ± 0.239^a^	0.887 ± 0.174^a^	0.331 ± 0.070^a^	2.696 ± 0.276^a^	0.477 ± 0.129^a^
	Treatment	1.229 ± 0.208^a^	0.892 ± 0.308^a^	0.338 ± 0.114^a^	2.676 ± 0.403^a^	0.516 ± 0.173^a^
S3	Control	0.898 ± 0.161^a^	0.670 ± 0.121^a^	0.228 ± 0.040^a^	2.930 ± 0.103^a^	0.297 ± 0.047^a^
	Treatment	1.195 ± 0.327^a^	0.883 ± 0.230^a^	0.313 ± 0.099^a^	2.863 ± 0.183^a^	0.507 ± 0.233^a^
S4	Control	1.165 ± 0.204^a^	0.863 ± 0.153^a^	0.303 ± 0.054^a^	2.855 ± 0.167^a^	0.589 ± 0.046^a^
	Treatment	1.082 ± 0.210^a^	0.794 ± 0.170^b^	0.288 ± 0.040^a^	2.733 ± 0.233^a^	0.649 ± 0.054^a^
S5	Control	0.964 ± 0.388^a^	0.701 ± 0.289^b^	0.263 ± 0.102^a^	2.672 ± 0.292^a^	0.340 ± 0.114^a^
	Treatment	1.246 ± 0.426^a^	0.927 ± 0.324^a^	0.318 ± 0.103^a^	2.902 ± 0.145^a^	0.480 ± 0.090^a^
S6	Control	1.443 ± 0.144^a^	1.070 ± 0.121^a^	0.373 ± 0.025^a^	2.865 ± 0.170^a^	0.541 ± 0.041^a^
	Treatment	0.948 ± 0.147^b^	0.714 ± 0.109^b^	0.234 ± 0.041^b^	3.070 ± 0.254^a^	0.577 ± 0.113^a^
S7	Control	0.958 ± 0.200^a^	0.705 ± 0.136^a^	0.253 ± 0.064^a^	2.825 ± 0.210^a^	0.590 ± 0.093^a^
	Treatment	1.205 ± 0.389^a^	0.892 ± 0.308^a^	0.313 ± 0.108^a^	2.869 ± 0.107^a^	0.703 ± 0.070^a^
S8	Control	1.035 ± 0.353^a^	0.777 ± 0.273^a^	0.258 ± 0.081^a^	2.973 ± 0.176^a^	0.642 ± 0.145^a^
	Treatment	1.451 ± 0.351^a^	1.071 ± 0.271^a^	0.379 ± 0.084^a^	2.815 ± 0.210^a^	0.849 ± 0.091^a^
S9	Control	1.404 ± 0.141^a^	1.217 ± 0.294^a^	0.437 ± 0.162^a^	2.871 ± 0.319^a^	0.583 ± 0.049^a^
	Treatment	1.171 ± 0.537^a^	0.884 ± 0.397^a^	0.287 ± 0.140^a^	3.124 ± 0.228^a^	0.641 ± 0.030^a^
S10	Control	1.172 ± 0.299^a^	0.871 ± 0.222^a^	0.301 ± 0.081^a^	2.908 ± 0.290^a^	0.485 ± 0.067^a^
	Treatment	1.167 ± 0.130^a^	0.856 ± 0.102^a^	0.486 ± 0.350^a^	2.820 ± 0.049^a^	0.673 ± 0.148^a^
S11	Control	1.138 ± 0.214^a^	0.844 ± 0.169^a^	0.294 ± 0.045^a^	2.859 ± 0.181^a^	0.460 ± 0.059^b^
	Treatment	1.448 ± 0.746^a^	1.043 ± 0.491^a^	0.404 ± 0.256^a^	2.751 ± 0.374^a^	0.748 ± 0.150^a^
S12	Control	1.211 ± 0.273^a^	0.921 ± 0.197^a^	0.291 ± 0.076^a^	3.214 ± 0.236^a^	0.393 ± 0.099^a^
	Treatment	0.935 ± 0.155^a^	0.716 ± 0.110^a^	0.219 ± 0.045^a^	3.293 ± 0.173^a^	0.435 ± 0.221^a^
S13	Control	1.083 ± 0.367^a^	0.805 ± 0.270^a^	0.277 ± 0.099^a^	2.937 ± 0.250^a^	0.608 ± 0.181^a^
	Treatment	1.237 ± 0.141^a^	0.920 ± 0.087^a^	0.317 ± 0.056^a^	2.934 ± 0.267^a^	0.600 ± 0.231^a^
S14	Control	0.917 ± 0.149^a^	0.685 ± 0.102^a^	0.231 ± 0.048^a^	2.995 ± 0.211^a^	0.319 ± 0.124^a^
	Treatment	1.018 ± 0.496^a^	0.761 ± 0.363^a^	0.257 ± 0.133^a^	3.022 ± 0.228^a^	0.510 ± 0.165^a^
S15	Control	1.062 ± 0.350^a^	0.707 ± 0.199^a^	0.241 ± 0.078^a^	2.941 ± 0.057^a^	0.468 ± 0.087^a^
	Treatment	1.111 ± 0.210^a^	0.837 ± 0.157^a^	0.295 ± 0.036^a^	3.065 ± 0.104^a^	0.574 ± 0.199^a^
S16	Control	1.046 ± 0.394^a^	0.769 ± 0.292^a^	0.277 ± 0.102^a^	2.765 ± 0.067^a^	0.546 ± 0.124^a^
	Treatment	1.244 ± 0.584^a^	0.897 ± 0.411^a^	0.383 ± 0.178^a^	2.623 ± 0.247^a^	0.634 ± 0.111^a^
S17	Control	1.047 ± 0.295^a^	0.772 ± 0.211^a^	0.275 ± 0.084^a^	2.823 ± 0.170^a^	0.564 ± 0.209^a^
	Treatment	1.218 ± 0.369^a^	0.911 ± 0.261^a^	0.263 ± 0.057^a^	3.015 ± 0.254^a^	0.591 ± 0.174^a^
S18	Control	1.126 ± 0.408^a^	0.844 ± 0.311^a^	0.283 ± 0.098^a^	2.974 ± 0.190^a^	0.586 ± 0.066^a^
	Treatment	1.266 ± 0.241^a^	0.951 ± 0.189^a^	0.271 ± 0.125^a^	3.015 ± 0.219^a^	0.687 ± 0.075^a^
S19	Control	1.239 ± 0.254^a^	0.907 ± 0.199^a^	0.332 ± 0.057^a^	2.715 ± 0.186^b^	0.517 ± 0.047^a^
	Treatment	0.923 ± 0.381^a^	0.680 ± 0.281^a^	0.302 ± 0.034^a^	2.818 ± 0.178^a^	0.542 ± 0.059^a^
S20	Control	0.636 ± 0.103^a^	0.468 ± 0.064^a^	0.168 ± 0.039^a^	2.830 ± 0.293^a^	0.289 ± 0.163^a^
	Treatment	0.886 ± 0.333^a^	0.657 ± 0.249^a^	0.221 ± 0.071^a^	2.862 ± 0.051^a^	0.565 ± 0.069^a^
S21	Control	1.085 ± 0.217^a^	0.803 ± 0.166^a^	0.282 ± 0.056^a^	2.850 ± 0.352^a^	0.414 ± 0.052^a^
	Treatment	1.187 ± 0.289^a^	0.871 ± 0.218^a^	0.411 ± 0.198^a^	2.774 ± 0.329^b^	0.417 ± 0.098^a^
S22	Control	1.144 ± 0.319^a^	0.841 ± 0.230^a^	0.303 ± 0.091^a^	2.797 ± 0.189^a^	0.597 ± 0.191^a^
	Treatment	1.668 ± 0.707^a^	1.196 ± 0.475^a^	0.369 ± 0.196^a^	2.650 ± 0.320^a^	0.677 ± 0.115^a^
S23	Control	0.967 ± 0.280^a^	0.722 ± 0.210^a^	0.245 ± 0.071^a^	2.947 ± 0.191^a^	0.439 ± 0.113^a^
	Treatment	1.020 ± 0.106^a^	0.751 ± 0.860^a^	0.275 ± 0.274^a^	2.789 ± 0.164^a^	0.498 ± 0.115^a^
S24	Control	1.088 ± 0.423^a^	0.800 ± 0.288^a^	0.288 ± 0.136^a^	2.882 ± 0.311^a^	0.464 ± 0.129^a^
	Treatment	1.109 ± 0.538^a^	0.820 ± 0.390^a^	0.289 ± 0.149^a^	2.890 ± 0.236^a^	0.506 ± 0.215^a^
S25	Control	1.022 ± 0.564^a^	0.755 ± 0.419^b^	0.267 ± 0.145^a^	2.817 ± 0.175^a^	0.471 ± 0.217^a^
	Treatment	1.228 ± 0.387^a^	0.907 ± 0.266^a^	0.321 ± 0.089^a^	2.816 ± 0.082^a^	0.649 ± 0.145^a^

The data in the table are the average values of each treatment, the results of physiological indices were expressed as mean ± standard deviation, different letters between control and treatment for each genotype indicate significant differences at *P<0.05* levels, through paired sample t-test. Chl, total chlorophyll content; Chl *a*, chlorophyll *a* content; Chl *b*, chlorophyll *b* content; Chl *a*/*b*, chlorophyll *a*/*b* ratio; Car, carotenoid content. The same below.

Chl *a* is the primary pigment responsible for energy conversion and is essential for photosynthesis. Our research indicated that, following drought treatment, Chl *a* levels increased in the majority of genotypes. The genotypes exhibiting the highest increases in Chl *a* content were Wutai No.1 (S22), Datong No.1 (S20) and Qinshui No.1 (S8) with increases of 42.21%, 40.38%, and 37.84% respectively. Chl *b*, another chlorophyll pigment found in the chloroplasts of plants and algae, plays a crucial supporting role in capturing additional wavelengths of light, thereby enhancing the efficiency of photosynthesis. In this study, Chl *b* content increased in 17 genotypes, while it decreased in 8. The largest increases were observed in Taigu No.1 (S10), Qinshui No.1 (S8), and Jiaocheng No.5 (S21), with increases of 61.46%, 46.90%, and 45.74%, respectively. The increase in Chl *b* content may contribute to the stabilization of the light-harvesting complex (LHC) under stress conditions^42,43^.

The Chl *a*/b ratio has been widely used as an indicator of drought resistance in plants^44^. Drought stress triggered the chlorophyll cycle-mediated conversion of Chl *b* to Chl *a*, leading to an elevated Chl *a*/*b* ratio^45^. In our experiment, we also observed an increase in the Chl *a/b* ratio across most genotypes. The genotypes showing the largest increases were Zezhou No. 2 (S9), Zuoquan No. 1 (S5), and Lishan No. 1 (S6), with increases of 8.81%, 8.61%, and 7.16%, respectively. Numerous studies have shown that terrestrial plants typically exhibit a Chl a/b ratio ranging from 2.0 to 3.5 under unstressed conditions. The observed ratios of 2.623 to 3.293 fall within this range, suggesting metabolic acclimation rather than structural impairment of the photosynthetic apparatus. In contrast, severe drought conditions typically elevate the Chl a/b ratio to values greater than 4.0^46^. This further highlights the mild to moderate nature of the drought stress in the present study, as well as the preservation of the photosynthetic apparatus.

Carotenoids are natural pigments found in most fruits and vegetables, plants, algae, and photosynthetic bacteria^47^. They serve multiple functions, including acting as antioxidants, phytohormones precursors, colorants, and essential components of the photosynthetic apparatus^48^. In addition, carotenoid plays an important role in plants’ resistance to abiotic stress. In this study, we observed a general increase in carotenoid content in *Hemerocallis fulva* leaves after drought stress, which is consistent with previous findings showing carotenoid accumulation in drought or high-salt environments in *Arabidopsis*^49^, *Salicornia europaea*^50^, sweet pepper^51^ and sweetpotato calli^52^.

**Fig. 2:**
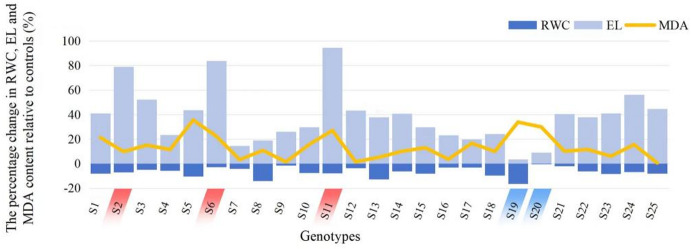
Percentage change in leaf relative water content (RWC), electrolyte leakage (EL), and malondialdehyde content (MDA) relative to control values in 25 *Hemerocallis fulva* genotypes under drought stress. RWC, leaf relative water content, EL, electrolyte leakage; MDA, malondialdehyde content. The same below.

The cell membrane is one of the first targets of many plant stresses and maintaining its integrity and stability under water deficit conditions is widely regarded as a key component of drought tolerance in plants^53^. MDA (malondialdehyde), a product of lipid peroxidation, serves as a widely used indicator of oxidative stress^54^. It has been demonstrated that MDA content increases in response to various abiotic stresses^55^. In this study, under drought conditions, the MDA content was higher in the leaves of all *Hemerocallis fulva* genotypes compared to the control (Fig. 2). Cell membrane injury induced by drought stress can be assessed by measuring electrolyte leakage^53^. In our study, electrolyte leakage increased in the leaves of all *Hemerocallis fulva* genotypes under drought stress. The genotypes exhibiting the smallest increases in electrolyte leakage were Datong No.1 (S20) and Jiaocheng No.1 (S19), with increases of 9.1% and 3.5%, respectively. In contrast, the genotypes showing the largest increases were Wutai No.2 (S2), Licheng No.6 (S6) and Jiaocheng No.4 (S11), with increases of 79.1%, 83.9% and 94.5%, respectively. These results suggest that drought stress causes membrane damage through lipid peroxidation, which subsequently leads to delayed growth and development (Table 2).

Relative water content (RWC) has been successfully employed to identify drought resistance in common bean^56^, maize^57^, and sunflower^58^, among various methods for evaluating water status in plants under drought conditions. In this study, compared with the control group, RWC of leaves in the drought treatment group exhibited a downward trend, with the largest decreases observed in Jiaocheng No.1(S19), Qinshui No.1(S8), and Jiaocheng No.3(S13), which were 16.49%, 14.15%, and 12.87% respectively.

These results of RWC, EL and MDA indicated that the *Hemerocallis fulva* genotypes exhibited different responses to drought stress, and different genotypes possessed different levels of drought resistance. Consequently, it was challenging to determine which genotypes were superior based on the above indices. Therefore, developing a more suitable method is essential for further evaluation.

### Principal component analysis

Assessing tolerance based on a single trait can only reflect the sensitivity of that trait towards drought stress. This can not reflect the comprehensive performance of a cultivar under drought stress. Therefore, it is insufficient to evaluate the drought of *Hemerocallis fulva* genotypes by a single indicator. PCA effectively diminishes the interference of redundant information by transforming a multitude of variables into a novel set of comprehensive and mutually exclusive components, thereby preserving the integrity of the original data^59^. In this research, after calculating the subordinate function values of the growth and physiological indices, PCA was utilized to comprehensive evaluate the drought resistance of *Hemerocallis fulva* genotypes. PCA revealed that the majority of the compositional information was encapsulated within the first three principal components. The individual contribution rates of these components were 32.91%, 23.92%, and 17.24%, respectively (Table 4). Consequently, the cumulative variance explained by their corresponding eigenvalues reached 74.06%, suggesting that these three principal components are sufficient to encapsulate the majority of the growth and physiological trait-related information pertinent to drought resistance.

**Table 4:** Eigenvalues and contribution rate of principal components.

Principal component	Eigenvalue	Contribution rate (%)	Cumulative contribution rate (%)
1	4.278	32.909	32.909
2	3.109	23.919	56.828
3	2.241	17.235	74.063
4	1.017	7.82	81.883
5	0.705	5.421	87.304
6	0.421	3.24	90.543
7	0.385	2.963	93.506
8	0.286	2.197	95.703
9	0.253	1.95	97.653
10	0.208	1.598	99.251
11	0.087	0.668	99.919
12	0.01	0.076	99.995
13	0.001	0.005	100

As shown in Table 5, the first principal component is characterized by four features that are acceptably correlated with it: plant height, canopy width, leaf length and leaf area. These four indices primarily reflect the growth conditions of plants under drought stress. Hence, the first principal component can be defined as a comprehensive index of plant growth. The second principal component is characterized by total chlorophyll content, chlorophyll *a* content, chlorophyll* b* content, and carotenoid content, and can thus be defined as a comprehensive index of photosynthetic pigments. The third principal component is characterized by two features: RWC and MDA content. RWC serves as an indicator of plant water status for screening drought resistance^60^, while MDA content reflects the degree of cell membrane injury. Accordingly, the third principal component can be defined as a comprehensive index of water status and cell membrane injury. These indices have been widely used in studies of *Iris*^22^, peanut^61^ and sesame^62^.

**Table 5:** Eigenvector matrix by principal component analysis.

Trait	Principal component		
	1	2	3
PH	0.909	− 0.097	− 0.079
CW	0.821	0.169	− 0.061
LL	0.939	0.027	0.05
LW	0.141	− 0.004	0.939
LA	0.697	− 0.009	0.678
Chl	− 0.179	0.887	− 0.116
Chl *a*	− 0.164	0.905	− 0.126
Chl *b*	− 0.125	0.467	0.132
Chl *a*/*b*	0.058	− 0.196	− 0.059
Car	0.186	0.809	0.225
RWC	0.135	0.029	− 0.764
EL	0.784	− 0.351	0.001
MDA	0.152	− 0.112	− 0.663

### Membership function analysis

The membership function values (μ) of different *Hemerocallis fulva* genotypes were obtained using the membership function method (Table 6). For example, based on the first principal component, the maximum membership function value was observed to be 1 for Datong No.1(S20), and the minimum value was 0 for Licheng No.1(S23). This indicates that Datong No.1 has the strongest drought tolerance, whereas Licheng No.1 shows the weakest.

**Table 6:** F values and D values of drought resistance evaluation for 25 *Hemerocallis fulva* genotypes.

Genotypes	Composite score (F)			Membership function values(μ)			D value(D)	Order
	F(X_1_)	F(X_2_)	F(X_3_)	μ(X_1_)	μ(X_2_)	μ(X_3_)		
S1	2.894	1.057	1.024	0.658	0.474	0.861	0.478	2
S2	1.997	0.985	− 0.141	0.424	0.447	0.405	0.316	13
S3	1.827	0.935	− 0.270	0.380	0.429	0.355	0.289	17
S4	3.075	0.787	0.257	0.705	0.374	0.561	0.418	3
S5	2.406	0.767	− 0.705	0.531	0.367	0.185	0.294	16
S6	2.723	0.210	0.319	0.613	0.161	0.585	0.341	10
S7	1.894	1.141	− 0.484	0.397	0.505	0.271	0.298	15
S8	3.403	2.182	0.535	0.790	0.890	0.670	0.588	1
S9	2.310	1.075	− 0.481	0.505	0.481	0.272	0.328	11
S10	2.011	1.432	0.646	0.428	0.613	0.713	0.410	4
S11	0.606	2.212	0.720	0.062	0.901	0.742	0.364	8
S12	1.867	− 0.225	− 1.162	0.390	0.000	0.006	0.129	24
S13	0.701	1.083	0.148	0.087	0.484	0.518	0.234	20
S14	1.329	0.438	0.183	0.250	0.245	0.532	0.233	21
S15	3.098	0.549	− 0.208	0.711	0.286	0.379	0.368	7
S16	0.917	1.394	− 0.338	0.143	0.599	0.328	0.247	19
S17	2.030	0.959	0.260	0.433	0.438	0.562	0.344	9
S18	1.907	1.131	− 1.178	0.401	0.502	0.000	0.252	18
S19	2.878	0.022	1.380	0.654	0.091	1.000	0.409	5
S20	4.209	− 0.150	− 0.241	1.000	0.028	0.366	0.399	6
S21	1.583	0.751	− 0.652	0.316	0.361	0.205	0.226	22
S22	0.879	2.479	− 0.861	0.133	1.000	0.124	0.304	14
S23	0.368	0.387	− 0.863	0.000	0.226	0.123	0.075	25
S24	0.977	0.577	0.319	0.158	0.297	0.585	0.224	23
S25	1.442	1.193	0.293	0.279	0.524	0.575	0.316	12
Weights				0.329	0.239	0.172		

In accordance with the PCA mathematical model, the standardized score value of the principal component, termed the composite score (F value), was computed. As shown in Table 6, Datong No.1 (S20) recorded the highest score (4.209) for the first principal component, reflecting minimal growth inhibition following drought treatment. Wutai No.1 (S22) ranked highest in the second principal component (2.479), suggesting elevated levels of photosynthetic pigments under drought conditions. Jiaocheng No.1 (S19) achieved the highest score (1.380) for the third principal component, indicating its capacity to maintain leaf relative water content and mitigate subsequent cell membrane damage.

Based on the contribution rate of each drought-resistant trait index, the weights of each principal component were calculated. The weights of the three principal components were estimated to be 0.329, 0.239 and 0.172, respectively. To comprehensively evaluate the drought resistance of *Hemerocallis fulva* genotypes, the comprehensive evaluation value (D value) was obtained by integrating the weights of three principal components to rank the drought tolerance (Table 6). A higher D value indicates a greater contribution of the corresponding component to drought resistance, suggesting stronger drought resistance. Table 6 demonstrates that Qinshui No.1(S8) and Taiyuan No.1(S1) exhibited the highest drought resistance, while Licheng No.1(S23) and Licheng No.3(S12) displayed the lowest drought resistance. Consequently, based on the D values, the drought resistance of the 25 *Hemerocallis fulva* genotypes was ranked accordingly.

### Clustering analysis

**Fig. 3:**
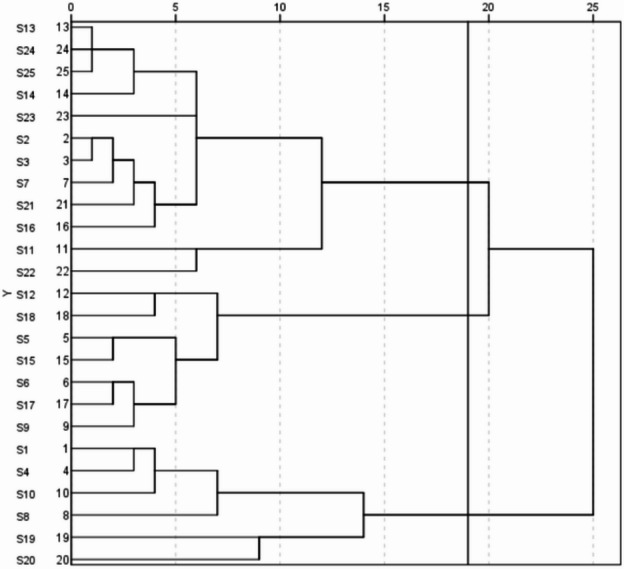
Cluster analysis diagram of 25 *Hemerocallis fulva* genotypes based on F value (the black line is the dividing line).

Using the method of the connection between groups, the 25 *Hemerocallis fulva* genotypes were clustered into three groups with a genetic distance of 19 in hierarchical cluster analysis (Fig. 3). Six genotypes were placed in Group I, including S20, S19, S8, S10, S4 and S1. This group is designated as the drought-resistant group, indicative of superior drought resistance. Group II consisted of 7 genotypes, identified by the serial numbers S9, S17, S6, S15, S5, S18 and S12 is classified as the medium drought-resistance group. The remaining twelve genotypes constitute Group III, which is characterized as the low drought resistance group.

**Fig. 4:**
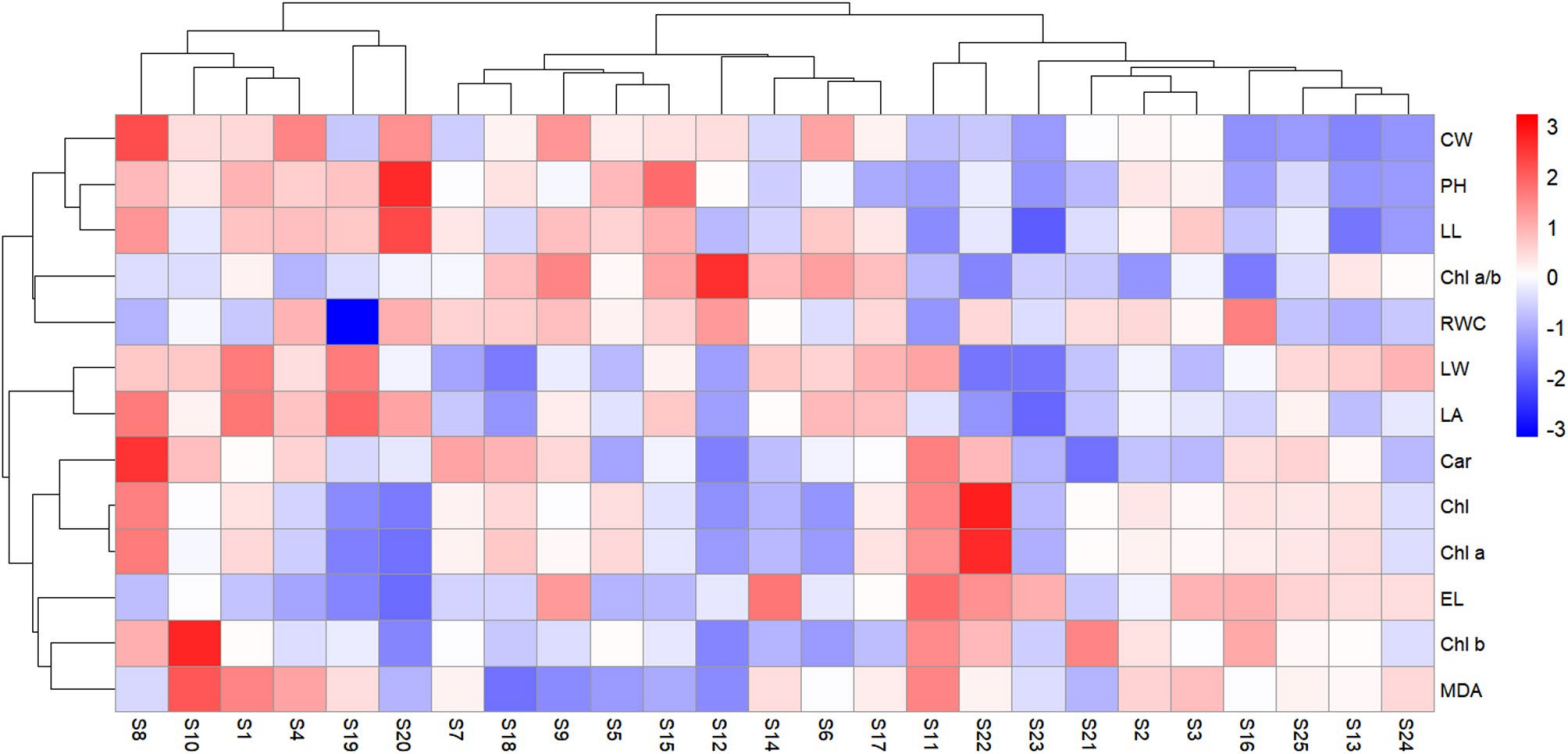
Cluster heat map analysis summarizing the responses of growth traits and physiological parameters to different genotypes of *Hemerocallis fulva* under drought stress. Each row in the heat map represents a specific index, and each column represents an individual *Hemerocallis fulva* genotype. The color intensity indicates the relative level of a index within a genotype, with red indicating a high level and green indicating low level.

On the other hand, a heat map analysis was performed for a better understanding of the variations in growth traits and physiological parameters among different *Hemerocallis fulva* genotypes under drought stress conditions (Fig. 4). Genotypes S8, S10, S1, S4, S19 and S20 constituted one cluster, designated as the drought-resistant group. Another cluster, characterized as the medium drought-resistance group, included genotypes S7, S18, S9, S5, S15, S12, S14, S6 and S17. The remaining 10 genotypes formed a third distinct cluster, categorized as the low drought-resistance group.

### Comprehensive evaluation of drought resistance

Based on the combined results of the comprehensive evaluation value (D value), cluster analysis diagram, and cluster heat map analysis, six highly drought-resistant genotypes were identified: S1, S4, S8, S10, S19, and S20, corresponding to Taiyuan No.1, Taiyuan No.2, Qinshui No.1, Taigu No.1, Jiaocheng No.1, and Datong No.1, respectively. This finding is consistent with the observed growth patterns of these genotypes. The phenotypic traits of these genotypes are provided in Table 7. These genotypes can be recommended as promising cultivars for drought-prone regions, such as the North China Plain and Mediterranean climates. Furthermore, they can be utilized in breeding programs, either through selective breeding or as donor parents, to further enhance drought tolerance in *Hemerocallis fulva*.

**Table 7:** Phenotypic traits of highly drought-resistant *Hemerocallis fulva* genotypes.

Material No	Material name	Chromosom number and ploidy	Plant type	Flower type	Flower color	Flowering phase	Petal type
S1	Taiyuan No.1	2n = 3x = 33	Medium plant	Large flower	Red series	Late	Double
S4	Taiyuan No.2	2n = 2x = 22	Medium plant	Small flower	Yellow series	Mid-season	Single
S8	Qinshui No.1	2n = 2x = 22	Small plant	Small flower	Orange series	Early	Single
S10	Taigu No.1	2n = 3x = 33	Large plant	Large flower	Red series	Mid-season	Single
S19	Jiaocheng No.1	2n = 3x = 33	Large plant	Large flower	Red series	Mid-season	Single
S20	Datong No.1	2n = 3x = 33	Large plant	Large flower	Yellow series	Mid-season	Single

Plant type: small plant (height < 40 cm), medium plant (height 40–60 cm), large plant (height > 60 cm). Flower type: micro flower (diameter < 7 cm), small flower (diameter 7–11 cm), large flower (diameter > 11 cm). Flowering phase: early (initial flowering period from early May to the end of May), mid-season (initial flowering period from early June to the end of July), late (initial flowering period from early August onwards).

### Correlation analysis between plant growth indices and physiological parameters

The correlation analysis between the plant growth indices with physiological parameters was shown in Table 8. A highly significant positive correlation was observed between the leaf area and plant height, canopy width, leaf length, and leaf width, with correlation coefficients of 0.548**, 0.538**, 0.735**, and 0.771**, respectively. Leaf width exhibited a highly significant negative correlation with leaf relative water content (RWC) (correlation coefficient = − 0.572**), and a highly significant positive correlation with malondialdehyde (MDA) content (correlation coefficient = 0.565**), a stable product of lipid peroxidation^63^. These findings suggest that, under drought stress, wider leaves in *Hemerocallis fulva* tend to have lower water retention and higher levels of oxidative stress, as evidenced by the MDA levels. Additionally, a strong positive correlation was observed between Chl *a* and Chl* b* contents. Total chlorophyll content was also highly significantly positively correlated with both Chl *a* and Chl* b*, as well as with the chlorophyll *a/b* ratio.

**Table 8:** Correlation coefficients of individual indices of 25 *Hemerocallis fulva* genotypes under drought stress.

Trait	PH	CW	LL	LW	LA	Chl	Chl *a*	Chl *b*	Chl *a*/*b*	Car	RWC	EL	MDA
PH	1.000												
CW	0.658**	1.000											
LL	0.837**	0.762**	1.000										
LW	0.026	0.062	0.171	1.000									
LA	0.548**	0.538**	0.735**	0.771**	1.000								
Chl	− 0.228	− 0.122	− 0.165	− 0.138	− 0.223	1.000							
Chl *a*	− 0.213	− 0.089	− 0.148	− 0.142	− 0.215	0.995**	1.000						
Chl *b*	− 0.204	− 0.148	− 0.204	0.112	− 0.069	0.603**	0.566**	1.000					
Chl *a*/*b*	0.131	0.283	0.096	− 0.010	0.048	− 0.455*	− 0.379	− 0.619**	1.000				
Car	0.075	0.223	0.152	0.188	0.223	0.579**	0.582**	0.371	− 0.259	1.000			
RWC	0.103	0.253	0.124	− 0.572**	− 0.371	0.006	0.017	− 0.151	0.135	− 0.124	1.000		
EL	− 0.717**	− 0.500*	− 0.621**	− 0.116	− 0.506**	0.388	0.373	0.201	− 0.092	0.091	0.033	1.000	
MDA	− 0.199	− 0.188	− 0.13	0.565**	0.272	0.169	0.124	0.453*	− 0.462*	0.232	− 0.370	0.225	1.000

**At the 0.01 level (two-tailed), the correlation is significant.

*At the 0.05 level (two-tailed), the correlation is significant.

### Correlation analysis between drought resistance and chromosome ploidy, and flower petals

The relationship between the D value of wild *Hemerocallis fulva* and chromosome ploidy, flower petals as well as physiology indices can be seen in Table 9. The results showed that there was no correlation between drought resistance with chromosome ploidy, or the number of flower petals in wild *Hemerocallis fulva*. A significant positive correlation was observed between the D value and the Chl *a*/*b* ratio (correlation coefficient = 0.494*), indicating that higher D values are associated with increased Chl *a/b* ratios. This finding may imply that changes in D value could be linked to alterations in chlorophyll composition, potentially affecting photosynthetic efficiency. In contrast, a highly significant negative correlation was found between the D value and electrolyte leakage (correlation coefficient = − 0.614**), implying that higher D values associated with reduced electrolyte leakage.

**Table 9:** Correlation coefficient (R) of the D value with chromosome ploidy and flower petals in wild *Hemerocallis fulva.*

	× chromosome ploidy	× flower petal	Chl	Chl *a*	Chl *b*	Chl *a*/*b*	Car	RWC	EL	MDA
D value	− 0.054	− 0.121	− 0.308	− 0.260	− 0.449	0.494*	0.127	0.101	− 0.614**	− 0.348

*P < 0.05; *P < 0.01.

## Conclusion

In summary, the results of our study revealed that the different *Hemerocallis fulva* genotypes displayed diverse responses to drought stress in growth and physiological traits. Thirteen individual growth and physiological indices of *Hemerocallis fulva* genotypes under drought stress were transformed into three principal components: a plant growth index, a photosynthetic pigments index, and a comprehensive water status and cell membrane damage index. The drought resistance of wild *Hemerocallis fulva* is not correlated with chromosome ploidy or the number of flower petals, but it is positively correlated with the chlorophyll *a*/*b* ratio. For drought-phenotyping in *Hemerocallis fulva*, photosynthetic pigments can be prioritised as the key trait; this shortens the screening time compared with field assays and is well-suited for resource-limited breeding programmes. Based on the combined results from the comprehensive evaluation value (D value), cluster analysis diagram, and cluster heat map analysis, the genotypes Taiyuan No.1, Taiyuan No.2, Qinshui No.1, Taigu No.1, Jiaocheng No.1 and Datong No.1 were classified into the drought-resistant group, suggesting that these six *Hemerocallis fulva* genotypes possess the highest drought resistance capability. These can be used by breeders for the genetic improvement of drought resistance in other *Hemerocallis fulva* genotypes and to provide greening plants for landscape architecture construction in arid area. Our results bridge the gap between phenotypic evaluation and breeding application, providing a validated, end-to-end pipeline for the development of drought-tolerant *Hemerocallis fulva* cultivars that is readily actionable for both academic and commercial breeders. Additionally, our study will provide a reasonable method for evaluating drought resistance in *Hemerocallis fulva* and other plants.

## Data Availability

All data are presented in the article, and can be requested from the corresponding author if required.
